# Process improvement decreases no show rates for pediatric cardiac MR imaging with anesthesia

**DOI:** 10.1186/1532-429X-15-S1-P283

**Published:** 2013-01-30

**Authors:** Charles Upshaw, Rebecca B Lee, Andrew L Rivard

**Affiliations:** 1 University of Mississippi Medical Center, Jackson, MS, USA

## Background

Coordination of pediatric imaging and anesthesia is a complex logistical challenge involving anesthesiologists, nurses, anesthetists, nurse practitioners, PACU nurses, radiologists, technologists and notwithstanding schedulers and receptionists. Parents often can be stressed, due to the child's NPO status and/or physical condition. Driving to an unfamiliar hospital and transportation difficulties can lead to an appointment no-show. We identified a high no-show rate in the formation of a pediatric cardiac imaging program and instituted process improvement changes to improve attendance in an academic center with a predominately rural population.

## Methods

Our 1st step was to eliminate the automated telephone system which was programmed to call the evening prior to anesthesia appointment. This eliminated erroneous automated phone calls if the appointment had been rescheduled. Our 2nd step included packet of instructions sent to the parent including the following: (1) an "Engagement" letter with instructions to arrive at the registration time (not the appointment time ~30 minutes later); (2) a cardiac MR exam information letter; (3) specific anesthesia diet instructions with the exact time (hh:mm) for solids/liquids and/or NPO status; (4) a letter of hospital location and parking directions, including arrows and pictures. This packet is sent by the schedulers to a physical mailing address of the parent. The 3rd step combined two separate phone calls by anesthesia and radiology into one - for a single point of verbal contact and instructions done the day prior to the exam. Data for 12 consecutive months of no-shows were collected manually by chart review. Patients who arrived late were considered to have attended appointments. Those rescheduled before they scheduled exam were defaulted to the next appointment.

## Results

The no-show rate for scheduled imaging studies utilizing pediatric anesthesiology and cardiac MR was 0% (84 exams). In comparison, the no-show rate of non-cardiac pediatric MR exams with anesthesia was 8% (2052 exams). An informal review revealed that anonymous automated telephone calls were not answered to association with hospital bill collecting. Also messages were generally not retrieved because of pre-paid mobile phone plans or disconnected land lines - thus leaving the US postal service as the sole mode of definitive communication.

## Conclusions

A considerable amount of effort goes into a pediatric cardiac MRI exam with anesthesia and our results support a process to improve attendance by 3 steps. We eliminated unnecessary and confusing messages by focusing efforts to communicate meaningful written and verbal information - before the scheduled appointment.

## Funding

None.

